# NLRX1 negatively modulates type I IFN to facilitate KSHV reactivation from latency

**DOI:** 10.1371/journal.ppat.1006350

**Published:** 2017-05-01

**Authors:** Zhe Ma, Sharon E. Hopcraft, Fan Yang, Alex Petrucelli, Haitao Guo, Jenny P-Y Ting, Dirk P. Dittmer, Blossom Damania

**Affiliations:** 1 Lineberger Comprehensive Cancer Center, University of North Carolina, Chapel Hill, North Carolina, United States of America; 2 Department of Microbiology and Immunology, University of North Carolina, Chapel Hill, North Carolina, United States of America; University of Southern California, UNITED STATES

## Abstract

Kaposi’s sarcoma-associated herpesvirus (KSHV) is a herpesvirus that is linked to Kaposi’s sarcoma (KS), primary effusion lymphoma (PEL) and multicentric Castleman’s disease (MCD). KSHV establishes persistent latent infection in the human host. KSHV undergoes periods of spontaneous reactivation where it can enter the lytic replication phase of its lifecycle. During KSHV reactivation, host innate immune responses are activated to restrict viral replication. Here, we report that NLRX1, a negative regulator of the type I interferon response, is important for optimal KSHV reactivation from latency. Depletion of NLRX1 in either iSLK.219 or BCBL-1 cells significantly suppressed global viral transcription levels compared to the control group. Concomitantly, fewer viral particles were present in either cells or supernatant from NLRX1 depleted cells. Further analysis revealed that upon NLRX1 depletion, higher IFNβ transcription levels were observed, which was also associated with a transcriptional upregulation of JAK/STAT pathway related genes in both cell lines. To investigate whether IFNβ contributes to NLRX1’s role in KSHV reactivation, we treated control and NLRX1 depleted cells with a TBK1 inhibitor (BX795) or TBK1 siRNA to block IFNβ production. Upon BX795 or TBK1 siRNA treatment, NLRX1 depletion exhibited less inhibitory effects on reactivation and infectious virion production, suggesting that NLRX1 facilitates KSHV lytic replication by negatively regulating IFNβ responses. Our data suggests that NLRX1 plays a positive role in KSHV lytic replication by suppressing the IFNβ response during the process of KSHV reactivation, which might serve as a potential target for restricting KSHV replication and transmission.

## Author summary

Kaposi’s sarcoma-associated herpesvirus (KSHV) is linked to a number of different human cancers, including Kaposi’s sarcoma (KS), primary effusion lymphoma (PEL) and multicentric Castleman’s disease (MCD). KSHV predominantly establishes life-long latency in the infected host. Lytic reactivation from latency is critical for KSHV survival and replication. During KSHV reactivation from latency, host innate immune responses are activated to restrict viral replication. Here, we report that NLRX1, a negative regulator of the type I interferon response, is important for optimal KSHV reactivation and subsequent lytic replication.

## Introduction

Kaposi’s sarcoma-associated herpesvirus (KSHV), also known as human herpesvirus 8 (HHV-8), is a linear double-stranded DNA virus. It is causally linked with Kaposi’s sarcoma (KS), primary effusion lymphoma (PEL) and multicentric Castleman’s disease (MCD) [1, 2]. In the majority of KS lesion cells or PEL cells, KSHV maintains latent infection, where KSHV lytic gene expression can only be detected in a small subset of cells [3]. While latent infection is important for maintaining the viral reservoir, reactivation of KSHV from latent infection is important for viral particle production and transmission of KSHV [4, 5]. The balance between lytic and latent infection is critical for KSHV pathogenicity. During reactivation, many cellular signaling pathways are activated and regulated by both host and viral proteins. For example, KSHV infection has been shown to trigger innate immune responses through pattern recognition receptors (PRRs) that recognize pathogen-associated molecular patterns (PAMPs), which results in interferon and pro-inflammatory cytokine production (reviewed in [6]). KSHV encodes multiple proteins that modulate host immune responses to facilitate viral replication [7–11]. However, a better understanding of the role of cellular genes in viral reactivation is also critical for understanding the pathogenesis associated with KSHV.

NLRX1 (also known as CLR11.3 and NOD9) is a member of nucleotide-binding domain, leucine-rich repeat-containing protein family (also known as NOD-like receptors, NLRs) [12–14]. NLRs were originally widely studied as sensors or receptors of inflammasome signaling, but were recently reported to regulate type I interferon production as well (reviewed in [15, 16]). NLRX1 was identified as a negative regulator of RIG-I like receptor (RLR) dependent type I interferon production [12]. NLRX1 localizes to the mitochondria and associates with MAVS (also known as Cardif, VISA and IPS-1), a mitochondria-localized adaptor downstream of RIG-I, to disrupt cytosolic RNA induced type I interferon responses. Depletion of NLRX1 led to enhanced antiviral responses [12]. Notably, NLRX1 was also identified to facilitate HIV-1 primary infection through negative regulation of the cytosolic DNA sensing pathway. NLRX1 was shown to sequester the DNA-sensing adaptor STING from interaction with TBK-1, and therefore block sufficient type I interferon production. In a similar vein, NLRX1 deficient mice also exhibited stronger innate immune responses and thus reduced HSV1 replication [17]. In correlation with these findings, in SIV infection of rhesus monkeys, expression of NLRX1 was negatively correlated with a type I interferon signature [18]. Therefore, in the context of viral infection, NLRX1 might be a key factor in determining the battle between host and virus.

KSHV reactivation has been previously reported to activate MAVS-dependent cytosolic RNA sensing pathways. Blocking MAVS was shown to downregulate type I interferon and thus facilitate KSHV lytic replication [19]. Therefore, as a MAVS negative regulator, it is plausible that NLRX1 plays an important role in KSHV reactivation, and functions as a potential target for restricting KSHV transmission.

## Results

### NLRX1 is required for optimal KSHV reactivation

**Fig 1:**
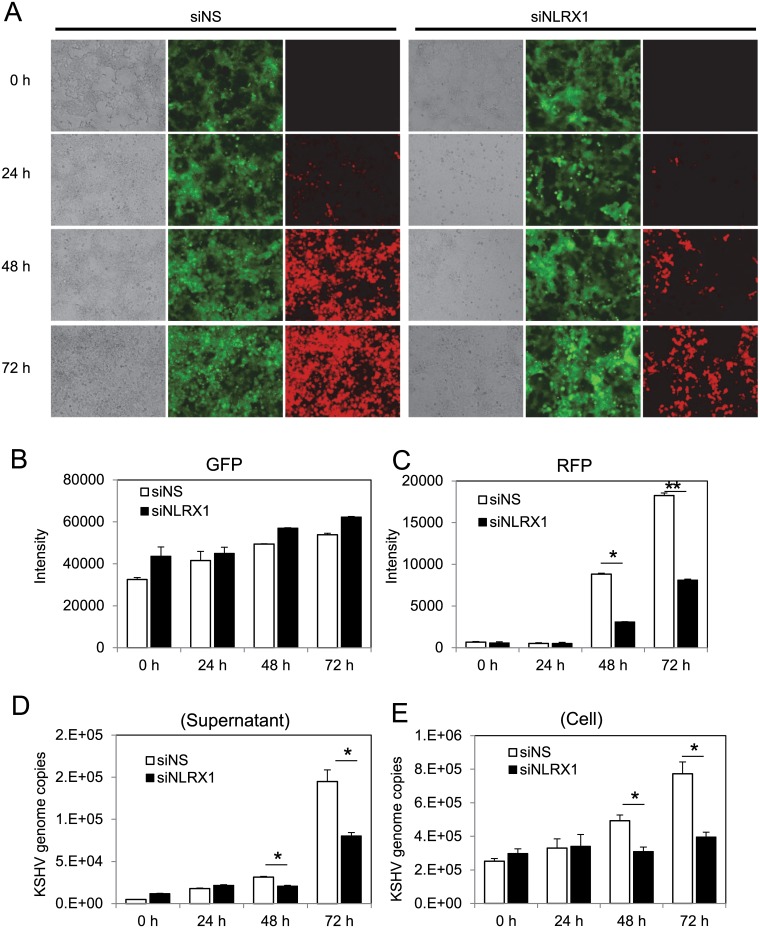
NLRX1 is required for optimal KSHV reactivation in iSLK.219 cells. (A) iSLK.219 cells were transfected with NS or NLRX1 siRNA for 48 hours and then treated with Dox for various time points. A representative image of a field of cells expressing GFP and RFP are shown at 0, 24, 48 and 72 hours post-Dox treatment. (B-C) Whole well GFP/RFP intensities were monitored and quantitated by a Clariostar plate reader. (D) KSHV genome copy numbers in the supernatants of reactivated iSLK.219 cells. (E) KSHV genome copy numbers in reactivated iSLK.219 cells. Data are presented as mean ± s.d. from at least three independent experiments. * indicates p<0.05. ** indicates p<0.01 by Student’s t-test.

**Fig 2:**
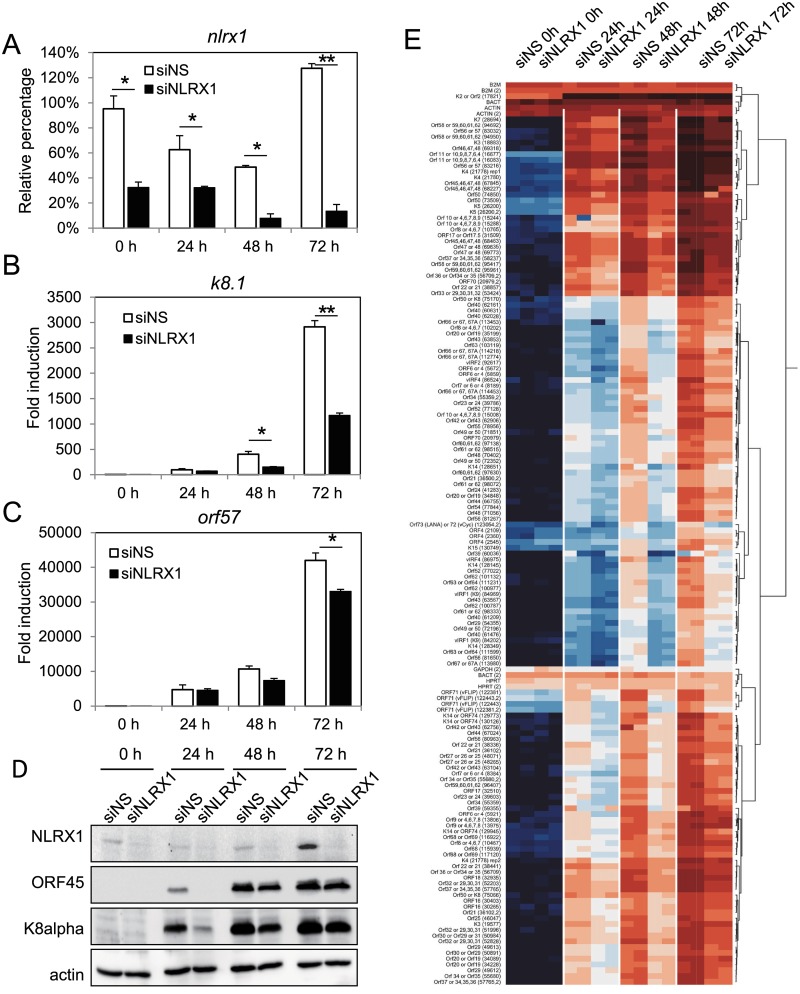
Loss of NLRX1 attenuates global KSHV gene expression upon reactivation. iSLK.219 cells were transfected with NS or NLRX1 siRNA for 48 hours and then treated with Dox for various time points. (A) qRT-PCR of NLRX1 in reactivated iSLK.219 cells. (B) qRT-PCR of K8.1 in reactivated iSLK.219 cells. (C) qRT-PCR of ORF57 in reactivated iSLK.219 cells. (D) Cells were harvested at the indicated time points for Western blot analysis of NLRX1, ORF45, and K8alpha. Actin was monitored as loading control. (E) iSLK.219 cells were treated as described in the text. RNA was extracted from duplicate samples and KSHV viral transcript levels were analyzed using a KSHV real-time qPCR-based whole genome array. mRNA levels of viral genes were normalized to the mRNA levels of multiple cellular housekeeping genes to yield dCT as a measure of relative expression. These were then subjected to unsupervised clustering. A heat map and dendrogram depicted by the brackets is shown. Higher transcript expression levels are indicated by red and lower expression levels by blue as shown in the key. Data are presented as mean ± s.d. from at least three independent experiments. *indicates p<0.05. ** indicates p<0.01 by Student’s t-test.

To investigate the role of NLRX1 as a potential restriction factor against KSHV lytic reactivation from latency, we utilized KSHV latently infected epithelial cells, iSLK.219 cells [20]. We used control siRNA or siRNA against NLRX1 to specifically deplete NLRX1 in iSLK.219 cells before reactivation. The iSLK.219 cells harbor a latent BAC16-KSHV with a constitutive GFP marker, a doxycycline (Dox)-inducible RTA, which is necessary and sufficient to induce lytic reactivation, and a RFP marker driven by the promoter of the KSHV lytic gene, PAN, which is activated during KSHV lytic replication. Therefore, GFP serves as a marker for infected cells while RFP serves as a marker for KSHV lytic replication. As shown in Fig 1A, while Dox successfully induced KSHV reactivation and lytic replication in both non-specific (NS) siRNA and NLRX1 siRNA transfected cells at each time point, there were lower percentages of RFP positive cells in the NLRX1 depleted samples. Moreover, while we did not observe significant GFP variation, RFP intensity quantitation showed significant inhibition of RFP intensity in the NLRX1 siRNA transfected samples compared to the control NS siRNA transfected samples. This suggests that KSHV lytic replication is suppressed in NLRX1 depleted cells (Fig 1B and 1C). We also tested a second NLRX1 siRNA (siNLRX1 #2) and examined its effect on viral reactivation by investigating RFP fluorescence. The results with the second NLRX1 siRNA was similar to the first siRNA (S2A and S2B Fig). We next monitored KSHV viral particles in the supernatant as well as in infected iSLK.219 cells from each group harvested at 24, 48 and 72 hours post reactivation. Consistent with RFP expression levels, we detected significantly fewer KSHV genomes in the NLRX1 siRNA transfected samples compared to control NS siRNA transfected samples as determined by KSHV genome copy number (Fig 1D and 1E). NLRX1 knockdown efficiency was checked by qRT-PCR (Fig 2A).

### NLRX1 is required for optimal KSHV gene transcription and expression upon reactivation

To determine the impact of NLRX1 on viral genes, we examined KSHV lytic gene expression. Knockdown efficiency of NLRX1 was monitored by qRT-PCR (Fig 2A, S2E Fig). NLRX1 deficient cells showed a reduced ability to induce lytic gene transcription (such as ORF57, vIRF1 and K8.1) than the control NS siRNA transfected cells (Fig 2B and 2C; S2C and S2D Fig). Both ORF45 and K8alpha lytic proteins were downregulated at 24, 48 and 72 hours post reactivation in the NLRX1 siRNA group (Fig 2D). Knockdown efficiency of NLRX1 was also monitored by immunoblot analysis (Fig 2D). To broadly profile KSHV viral gene expression during reactivation in control versus NLRX1 depleted samples, we also performed KSHV whole genome transcriptional profiling. As seen in Fig 2E, Dox treatment successfully induced KSHV gene expression, and depletion of NLRX1 led to a suppression and delay of viral genome transcription at each time point that we tested, which correlates well with our qRT-PCR data (Fig 2E). We also monitored viral gene transcription at later time points, and we observed a smaller difference between the siNS and siNLRX1 groups at later time points (S3A–S3C Fig).

### Loss of NLRX1 results in increased type I interferon responses

We next probed for the mechanism by which NLRX1 depletion restricts KSHV lytic reactivation from latency. In order to rule out the possibility that siNLRX1 affects the induction of RTA, we tested if siNLRX1 would affect RTA mRNA transcription induced by Dox. Because RTA transcription in iSLK.219 cells can be both affected by Dox and KSHV reactivation, we used iSLK.RTA cells without KSHV infection to test if siNLRX1 would affect Dox induced RTA. As shown in S3D and S3E Fig, knockdown of NLRX1 did not attenuate RTA mRNA transcription.

**Fig 3:**
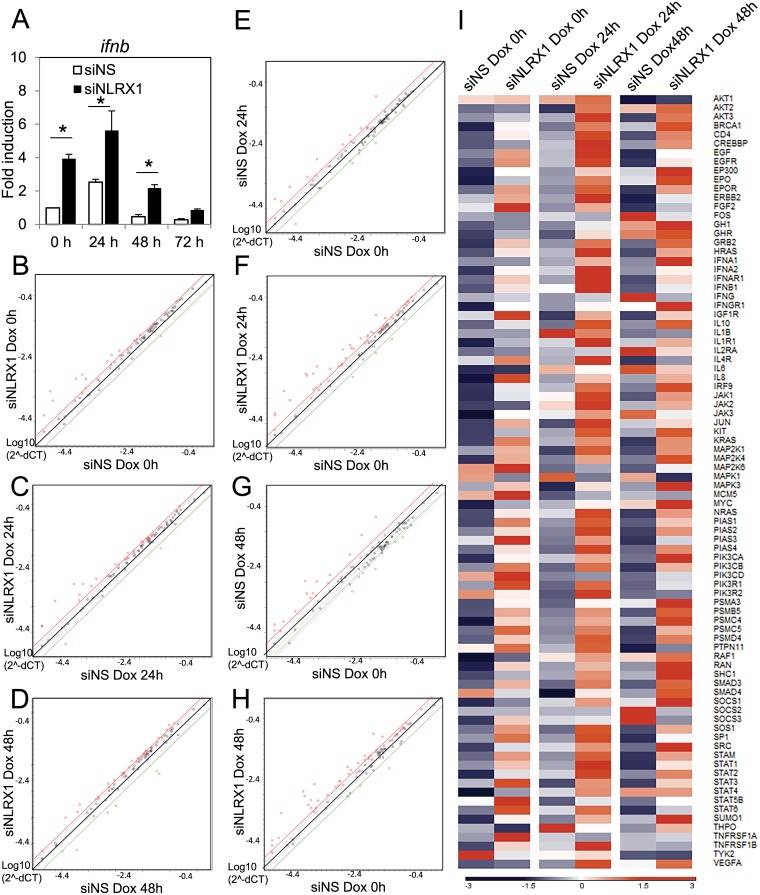
NLRX1 depletion results in enhanced interferon responses upon KSHV reactivation. iSLK.219 cells were transfected with NS or NLRX1 siRNA for 48 hours and then treated with Dox for various time points. (A) IFNβ mRNA levels were monitored by qRT-PCR. (B-D) RNA was extracted from duplicate samples and various JAK/STAT related mRNA levels were analyzed using a JAK/STAT real-time qPCR-based gene array (details are listed in S1 Table). mRNA levels of genes were normalized to the mRNA levels of human β-actin to yield dCT as a measure or relative expression without clustering. Scatter plot comparison of relative mRNA level at 0 (B), 24 (C) and 48 (D) hours between NLRX1 siRNA treated cells and NS siRNA treated cells. (E-H) Scatter plot of relative mRNA level at 24 and 48 hours. NS siRNA group at 0 hours were set as normalization control. (I) Heat map of the JAK/STAT microarray. Relative Z-scores of each gene level was calculated by subtracting mean values for each individual gene, and then dividing by each gene standard deviation. Higher Z-scores are indicated by red, lower levels by blue as shown in the key. Data are presented as mean ± s.d. from at least three independent experiments. *indicates p<0.05. ** indicates p<0.01 by Student’s t-test.

Since NLRX1 has been reported to negatively regulate type I interferon, we then investigated if NLRX1 altered type I interferon responses upon KSHV reactivation. As seen in Fig 3A, loss of NLRX1 did result in stronger *ifnb* transcriptional activity. IFNβ is known to activate the JAK/STAT pathway to induce ISGs for effective antiviral responses against viruses. To investigate this further, we next tested whether the upregulated *ifnb* transcription level in NLRX1 deficient cells led to the activation of the JAK/STAT pathway. We performed microarray analysis of genes in the JAK/STAT pathway using KSHV infected cells that were transfected with either control or NLRX1 siRNAs at timepoints of 0 hour, 24 hours and 48 hours post reactivation. We compared JAK/STAT responsive genes that were activated in cells transfected with NLRX1 and NS siRNA at each time point. At 0, 24 and 48 hours, many JAK/STAT pathway genes were induced at least 2 fold higher in NLRX1 depleted cells compared to NS siRNA transfected cells, indicating a higher potential for JAK/STAT pathway upregulation upon NLRX1 depletion (Fig 3B–3D). Moreover, as shown in Fig 3E and 3F, at 24 hours post Dox treatment, 16 genes were upregulated by 2 fold in the NS siRNA control samples while 48 genes were upregulated at least 2 fold in the in NLRX1 siRNA transfected samples. At 48 hours post Dox treatment, 20 genes were upregulated by more than 2 fold in the NS siRNA control samples while 46 genes were upregulated at least 2 fold in the NLRX1 siRNA transfected samples (Fig 3G and 3H). In summary, NLRX1 deficient cells induce a much wider variety of JAK/STAT pathway related genes at each time point tested in KSHV reactivated cells compared to the control cells. Fig 3I and S1 Table depicts the overall gene expression of all 84 JAK/STAT related genes tested. As shown, most genes exhibit a higher expression level in NLRX1 deficient cells compared to control cells at each time point tested. We noticed that many JAK/STAT related genes were upregulated in the absence of KSHV lytic reactivation. To eliminate that this is not due to an off target effect of the NLRX1 siRNA, we also tested an alternative NLRX1 siRNA (siNLRX1 #2) to monitor the activation of the JAK/STAT pathway before KSHV was reactivated. As shown in S4A–S4D Fig, we have also observed many upregulated genes. Specifically, of 57 upregulated genes in siNLRX1 #2 group, 54 of the genes were also upregulated in siNLRX1 group, compared to the siNS group. We also transiently overexpressed NLRX1 in iSLK.219 cells to explore the role of NLRX1 in KSHV reactivation. Consistent with the siRNA experiments, we detected attenuated levels of IFNβ mRNA and increased levels of viral ORF57 mRNA (S1A and S1B Fig). NLRX1 mRNA levels were monitored by qRT-PCR (S1C Fig).

### NLRX1 negatively regulates MAVS-dependent type I interferon responses

**Fig 4:**
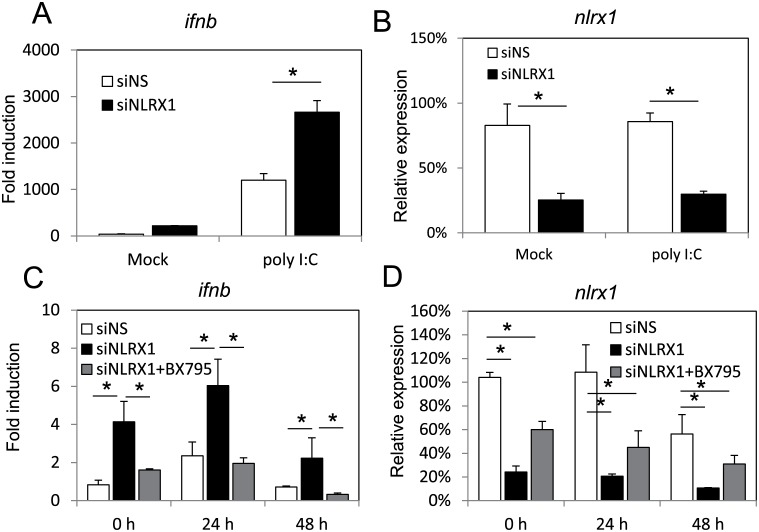
NLRX1 negatively regulates MAVS-dependent type I interferon response. (A) iSLK.219 cells were transfected with NS or NLRX1 siRNA for 72 hours. Cells were then transfected with poly I:C at 2 μg/μl for 4 hours before being harvested. RNA was subjected to IFNβ qRT-PCR analysis. (B) NLRX1 knockdown efficiency was monitored by qRT-PCR. (C) iSLK.219 cells were transfected with NS, NLRX1 siRNA for 72 hours. 1 μM BX795 was then added to one set of NLRX1 siRNA transfected samples 6 hours before the cells were reactivated with Dox for 0, 24 and 48 hours. IFNβ mRNA was monitored by qRT-PCR. (D) NLRX1 knockdown efficiency was monitored by qRT-PCR.

NLRX1 was identified as a negative regulator of MAVS. Therefore, in order to further detail the mechanism by which NLRX1 facilitates KSHV reactivation, we looked at IFNβ regulation by MAVS. MAVS has previously been shown to play a role in limiting KSHV reactivation. Therefore, we hypothesized that NLRX1 blocks MAVS to attenuate type I interferon responses in KSHV-infected cells, and thus facilitates KSHV reactivation. To prove this, we first tested the integrity and functionality of the MAVS signaling pathway. As shown in Fig 4A, we transfected poly I:C into KSHV infected iSLK.219 cells and successfully triggered MAVS dependent IFNβ induction, which is indicative of a functional RLR pathway. Moreover, NLRX1 depletion in these cells resulted in higher induction of IFNβ, suggesting NLRX1 blocks MAVS signaling in KSHV-infected iSLK.219 cells without reactivation. NLRX1 knockdown efficiency was monitored by qRT-PCR (Fig 4B). We also tested if poly I:C, an activator of the MAVS pathway, could mimic the effect of NLRX1 knockdown to inhibit KSHV reactivation. As shown in S5A–S5F Fig, poly I:C induced IFNβ during reactivation, and this correlated with inhibition of KSHV reactivation as determined by RFP fluorescence and viral lytic gene expression.

To further test if NLRX1 blocks MAVS signaling in KSHV-infected cells upon reactivation, we used BX795, an inhibitor of TBK1, which acts directly downstream of MAVS. As shown in Fig 4C, when we treated cells with NLRX1 siRNA and BX795, we observed significant inhibition of IFNβ compared to the NLRX1 siRNA and vehicle only treated group at 24 and 48 hours post reactivation. IFNβ levels in the NLRX1 siRNA and BX795 treated group were similar to that of the NS siRNA treated group. NLRX1 knockdown efficiency was monitored by qRT-PCR (Fig 4D). To eliminate the possibility that NLRX1 directly acts as a negative regulator of IRF3, we tested whether NLRX1 could modulate IFNβ promoter luciferase activation by an IRF3 super activator (SA). As shown in S1D–S1F Fig, NLRX1 overexpression inhibited dRIG-I- or MAVS- dependent activation of the IFNβ promoter, but the IRF3(SA) activated IFNβ promoter activation was not affected. These data suggest that NLRX1 inhibits MAVS in KSHV-infected cells and supports KSHV lytic replication.

### TBK1 inhibition by BX795 partially rescues KSHV reactivation and gene expression

**Fig 5:**
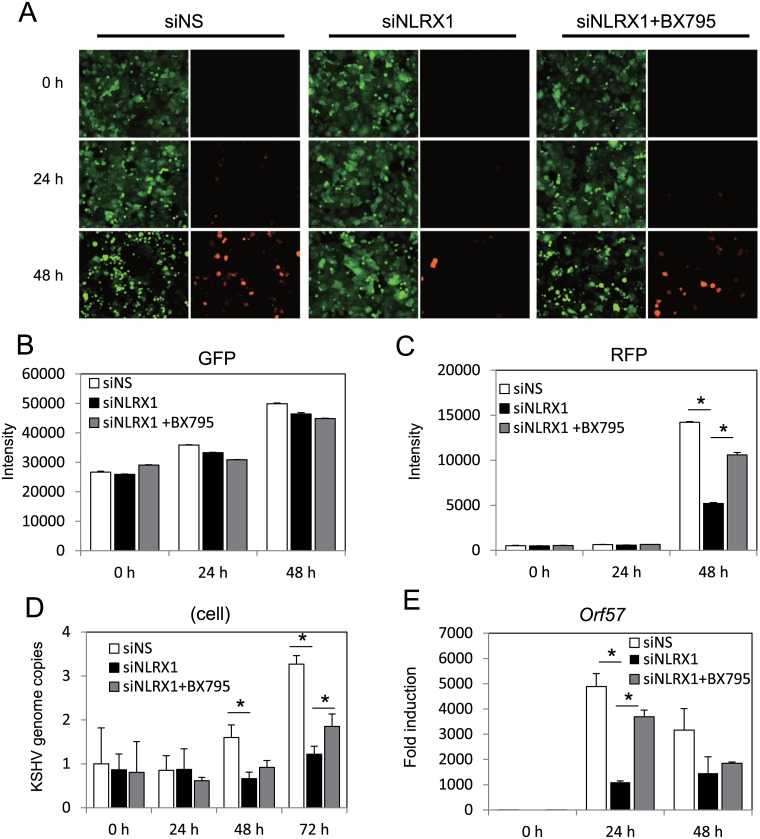
Block of TBK1 by BX795 partially rescues KSHV reactivation. iSLK.219 cells were transfected with NS, or NLRX1 siRNA for 72 hours. 1 μM BX795 was then added to one set of NLRX1 siRNA treated samples 6 hours before the cells were reactivated by Dox for 0, 24 and 48 hours. (A) A representative image of a field of cells expressing GFP and RFP are shown at 0, 24, and 48 hours post-Dox treatment. (B-C) Whole well GFP/RFP intensities were monitored and quantitated by Clariostar plate reader. (D) KSHV genome copy number in reactivated iSLK.219 cells. (E) qRT-PCR of ORF57 in reactivated iSLK.219 cells. Data are presented as mean ± s.d. from at least three independent experiments. * indicates p<0.05. ** indicates p<0.01 by Student’s t-test.

We next investigated whether BX795 inhibition of the MAVS-TBK1 node would rescue the effect of NLRX1 depletion on KSHV reactivation. As shown in Fig 5A, while NLRX1 depletion resulted in less RFP positive cells than the control NS siRNA group, BX795 treatment partially rescued the block of lytic replication. RFP intensity quantitation also showed significant inhibition of RFP intensity in NLRX1 siRNA treated samples compared to the control group, and a subsequent increase of RFP intensity when the NLRX1 siRNA group was treated with BX795 (Fig 5C). No significant variations were observed in GFP intensity among groups (Fig 5B). We then monitored KSHV viral particles from the reactivated cells from each group harvested at 24, 48 hours and 72 hours post reactivation. We detected significantly fewer KSHV virions in cells transfected with NLRX1 siRNA compared to control NS siRNA samples, and a partial rescue of KSHV viral genomes upon BX795 treatment (Fig 5D). Similar patterns were observed when we monitored KSHV ORF57 gene transcription levels in reactivated iSLK.219 cells (Fig 5E). NLRX1 depletion led to a significant inhibition of ORF57 gene transcription, but this was partially rescued by BX795 treatment (Fig 5E).

### Knockdown of TBK1 by siRNA rescues KSHV reactivation and gene expression

**Fig 6:**
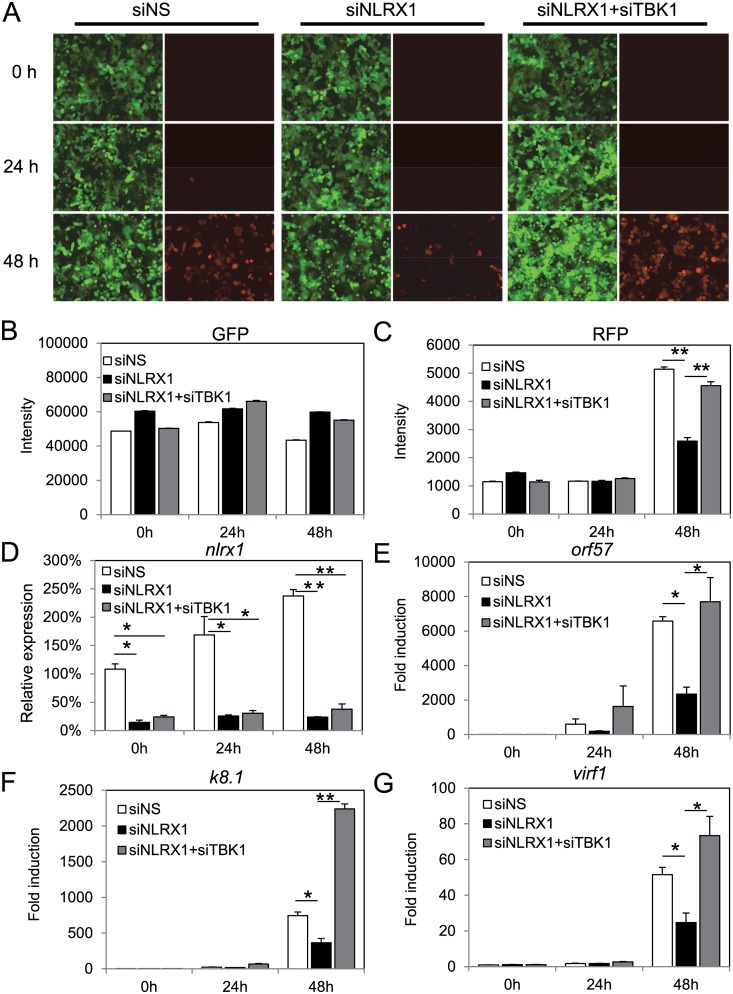
TBK1 siRNA rescues KSHV reactivation. iSLK.219 cells were transfected with NS, NLRX1 siRNA or NLRX1+TBK1 siRNAs for 48 hours. iSLK.219 were then reactivated with Dox for 0, 24 and 48 hours. (A) A representative image of a field of cells expressing GFP and RFP are shown at 0, 24, and 48 and hours post-Dox treatment. (B-C) Whole well GFP/RFP intensities were monitored and quantitated by a Clariostar plate reader. (D) NLRX1 knockdown efficiency was monitored by qRT-PCR. (E-G) qRT-PCR of ORF57, K8.1 and vIRF1 in reactivated iSLK.219 cells. Data are presented as mean ± s.d. from at least three independent experiments. * indicates p<0.05. ** indicates p<0.01 by Student’s t-test.

To further corroborate our experimental data obtained with BX795 treatment, we also utilized TBK1 specific siRNA. As shown in Fig 6A, while NLRX1 depletion resulted in fewer RFP positive cells than the control NS siRNA sample, siNLRX1+siTBK1 treatment rescued the block to KSHV lytic reactivation and replication. RFP intensity quantitation also showed significant inhibition of RFP intensity in NLRX1 siRNA treated samples compared to the control samples and a recovery of RFP intensity when the NLRX1 siRNA group was co-transfected with siNLRX1 and siTBK1 (Fig 6C). No significant variations were observed in GFP intensity among groups (Fig 6B). We also monitored multiple KSHV viral gene transcripts in reactivated iSLK.219 cells. As shown in Fig 6D–6G, NLRX1 depletion led to significant inhibition of ORF57, K8.1 and vIRF1 gene transcription, but they were all rescued when the cells were co-transfected with TBK1 siRNA. NLRX1 knockdown efficiency was monitored by qRT-PCR as shown in Fig 6D. We have also tested if TBK1 knockdown alone promoted KSHV replication by examining viral lytic gene transcription by qRT-PCR. As shown in S6A–S6D Fig, TBK1 knockdown resulted in elevated transcription of viral genes, such as *orf57*, *virf1* and *k8*.*1*.

### NLRX1 is required for optimal KSHV reactivation and viral gene expression in PEL

**Fig 7:**
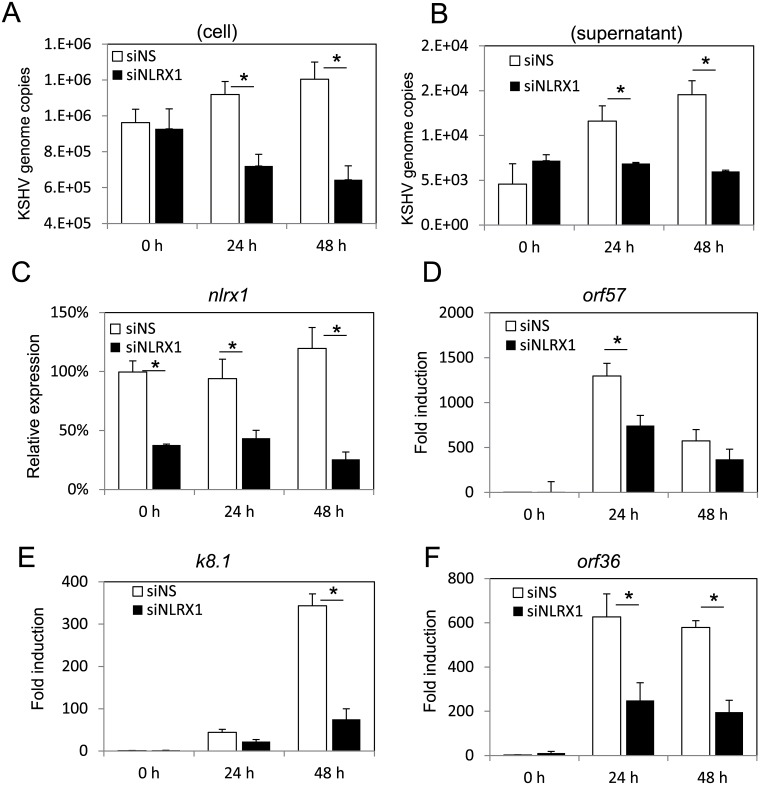
NLRX1 is required for optimal KSHV reactivation in BCBL-1 cells. BCBL-1 cells were transfected with NS or NLRX1 siRNA for 48 hours and then treated with TPA and sodium butyrate for various time points. (A) KSHV genome copy number in reactivated BCBL-1 cells. (B) KSHV genome copy number in the supernatants of reactivated BCBL-1 cells. (C) qRT-PCR of NLRX1 in reactivated BCBL-1 cells. (D) qRT-PCR of ORF57 in reactivated BCBL-1 cells. (E) qRT-PCR of K8.1 in reactivated BCBL-1 cells. (F) qRT-PCR of ORF36 in reactivated BCBL-1 cells. Data are presented as mean ± s.d. from at least three independent experiments. * indicates p<0.05. ** indicates p<0.01 by Student’s t-test.

We also investigated NLRX1’s role in KSHV infected PEL cells. BCBL-1 is a KSHV-infected B lymphoma cell line. We transfected NS or NLRX1 siRNA into BCBL-1 cells, and then induced lytic replication of KSHV by addition of TPA and sodium butyrate (NaB) as previously described [11]. The cells and supernatant were harvested at 0 hour, 24 hours and 48 hours post reactivation, and KSHV genome copy number was determined by qRT-PCR. As shown in Fig 7A and 7B, NLRX1 depletion resulted in significant inhibition of KSHV viral replication both in the cells and in the supernatant, as determined by the genome copy number. We also tested KSHV gene transcription levels in reactivated BCBL-1 cells transfected with NLRX1 siRNA or NS siRNA. NLRX1 depletion resulted in significant inhibition of lytic gene transcription such as ORF57 (Immediate early), ORF36 (early), and K8.1 (late) than NS siRNA transfected cells (Fig 7D–7F). NLRX1 knockdown efficiency was monitored by qRT-PCR as shown in Fig 7C. Furthermore, we also introduced another siRNA (siNLRX1 #2) and performed qRT-PCR assay to monitor the status of KSHV lytic genes in reactivated BCBL-1 cells. As shown in S7A–S7D Fig, we observed that both NLRX1 siRNAs modulated KSHV reactivation similarly in BCBL-1 cells.

### Loss of NLRX1 results in enhanced type I interferon responses in BCBL-1 cells

**Fig 8:**
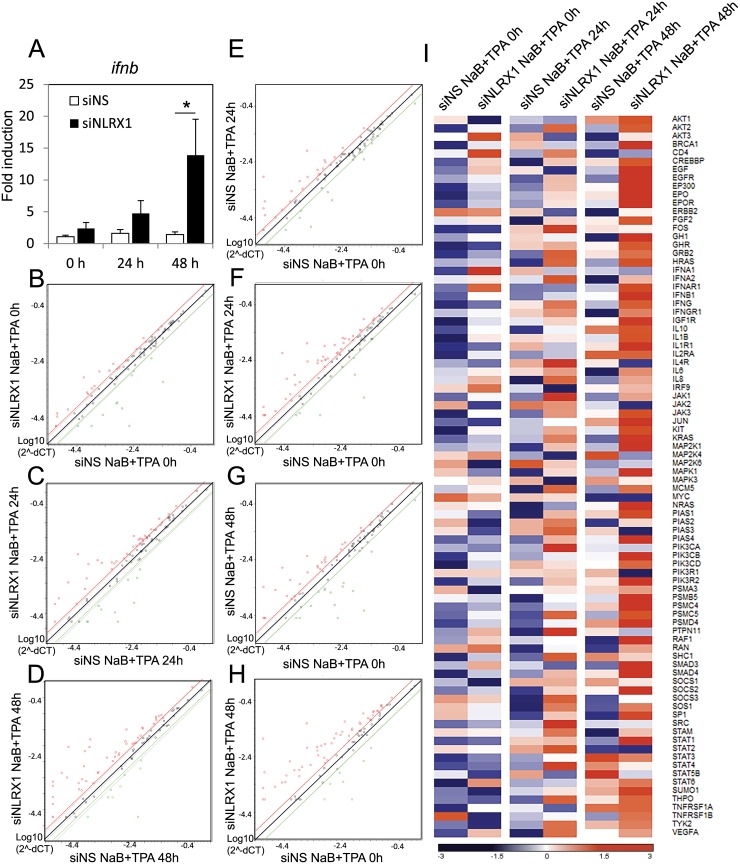
NLRX1 knock down results in enhanced interferon responses upon KSHV reactivation in BCBL-1 cells. BCBL-1 cells were transfected with NS or NLRX1 siRNA for 48 hours and then treated with TPA and sodium butyrate for various time points. (A) IFNβ mRNA in BCBL-1 cells was monitored by qRT-PCR. (B-D) RNA was extracted from duplicate samples and various JAK/STAT related mRNA levels were analyzed using a JAK/STAT real-time qPCR-based gene array. mRNA levels of viral genes were normalized to the mRNA levels of human β-actin to yield dCT as a measure or relative expression without clustering. Scatter plot comparison of relative mRNA level at 0 (B), 24 (C) and 48 (D) hours between NLRX1 siRNA treated cells and NS siRNA treated cells. (E-H) Scatter plot of relative mRNA level at 24 and 48 hours. NS siRNA group at 0 hours were set as the normalization control. (I) Heat map of JAK/STAT microarray. Relative Z-scores of each gene level was calculated by subtracting mean values for each individual gene, and then dividing by each gene standard deviation. Higher Z-scores are indicated by red, lower levels by blue as shown in the key. Data are presented as mean ± s.d. from at least three independent experiments. *indicates p<0.05. ** indicates p<0.01 by Student’s t-test.

Because NLRX1 is a negative regulator of IFNβ, we tested if NLRX1 blocked type I interferon responses upon KSHV reactivation. As seen in Fig 8A, NLRX1 depletion enhanced *ifnb* transcriptional activity compared to the NS siRNA group, confirming NLRX1’s role in restricting IFNβ. We then performed microarray analysis of the JAK/STAT pathway in BCBL-1 cells to explore NLRX1’s effect on KSHV reactivation. We compared genes that were activated in cells treated with NLRX1 siRNA or NS siRNA at 0, 24 and 48 hours post reactivation. At 0, 24 and 48 hours, a significant number of genes were induced at least 2 fold higher in NLRX1 siRNA transfected cells compared to NS siRNA transfected cells, indicating a higher potential of JAK/STAT pathway upregulation when NLRX1 is depleted in BCBL-1 cells (Fig 8B–8D). Moreover, at 24 hours post reactivation, 21 genes were upregulated by more than 2 fold in the siNS group while 41 genes in the siNLRX1 group were upregulated by 2 fold. At 48 hours post reactivation, 29 genes were upregulated by more than 2 fold in the siNS group while 49 genes in the siNLRX1 group were upregulated by more than 2 fold. These data suggest that NLRX1 deficient cells exhibited induction of a much wider variety of JAK/STAT pathway related genes at each time point tested compared to the control groups (Fig 8E–8H). Fig 8I and S2 Table summarizes the overall gene expression of all 84 JAK/STAT related genes tested. As shown, most genes exhibit higher expression levels in NLRX1 depleted cells compared to control cells at each time point tested.

## Discussion

KSHV reactivation from latency is a complex process for both virus and host, which is tightly regulated by a variety of signaling pathways. Efficient lytic replication of KSHV requires disruption of restrictive signaling pathways that keep the virus latent. This can be achieved by the action of either viral proteins or host proteins. For example, upon KSHV reactivation, cytosolic RNA and DNA dependent pathways were reported to be activated and type I interferon was produced to suppress viral replication [8, 19]. Previously, we have reported that in order to facilitate viral lytic replication, KSHV encodes multiple proteins to inhibit type I interferon production, such as vIRF1. vIRF1 knockdown in the context of viral reactivation can result in enhanced IFNβ production and insufficient reactivation [8]. While viral inhibitors of type I interferon are important for KSHV lytic replication, in this study, we focused on exploring the role of a host type I interferon inhibitor, NLRX1, during KSHV reactivation. We demonstrated that NLRX1 is required for optimal KSHV reactivation. NLRX1 deficiency in iSLK.219 cells led to enhanced type I interferon production, as well as global suppression of KSHV genome transcription activity, decreased level of lytic proteins, and attenuated virion production. A similar phenotype was observed in BCBL-1 cells as well, suggesting NLRX1 is critical for KSHV reactivation and subsequent replication in multiple cell lines. We noticed that viral genes clustered together based on their expression patterns following NLRX1 depletion. A future goal will be to further explore the differences among these different clusters to better understand the regulation of KSHV reactivation.

NLRX1 was previously reported to suppress the RIG-I-MAVS signaling pathway, which is triggered by cytosolic RNA [12]. It has also been previously reported that KSHV reactivation generates dsRNA intermediates that can trigger RIG-I-MAVS signaling in KSHV-infected cells [19]. As shown in our study, we demonstrate the functionality of the MAVS-dependent pathway in both KSHV-infected iSLK.219 and BCBL-1 cells. More importantly, NLRX1 negatively regulated MAVS-dependent IFNβ transcription before and after KSHV reactivation in both iSLK.219 and BCBL-1 cells, indicating that NLRX1 regulates KSHV reactivation through a MAVS-dependent pathway. Inhibition of type I interferon induction by the TBK1 antagonist, BX795, mitigated the effect of NLRX1 deficiency on KSHV lytic replication.

We have also explored TBK1’s effect on KSHV lytic reactivation. Although this is the first time that TBK1 was reported to be a negative regulator of KSHV lytic reactivation, TBK1 was previously reported as a restriction factor of RNA viruses, such as Newcastle Disease virus (NDV) and Sendai virus (SeV), by acting downstream of MAVS and positively regulating IFN responses [21, 22]. This correlates with our previously published results that MAVS play a negative role in KSHV reactivation [19]. Moreover, TBK1 also inhibits replication of HSV-1, an alpha herpesvirus [23].

Although NLRX1 plays an important role in KSHV reactivation, we did not observe significant upregulation or downregulation of NLRX1 at either the transcript or protein level during KSHV reactivation. Therefore, it is possible that NLRX1 serves as a steady-state negative regulator of type I interferon to facilitate KSHV reactivation. However, it is also plausible that NLRX1 may undergo some type of post translational modifications upon KSHV reactivation, which might further benefit KSHV lytic replication. A third possibility is that during lytic reactivation, a KSHV encoded protein(s) might also bind directly or indirectly to the NLRX1 signaling complex to regulate its function. In sum, we report for the first time that NLRX1 plays a pivotal role in modulating KSHV reactivation from latency.

## Materials and methods

### Cell culture, reagents, and antibodies

iSLK.219 (doxycycline-inducible SLK cells harboring latent rKSHV.219) (a kind gift from D. Ganem) were maintained in DMEM (Corning) supplemented with 10% FBS (Sigma), 1% penicillin and streptomycin (Corning), G418 (250 μg/ml) (Sigma), hygromycin (400 μg/ml) (Corning), and puromycin (10 μg/ml) (Corning). BCBL-1 cells (a kind gift from D. Ganem) were maintained in RPMI (Corning) medium supplemented with 20% FBS, 1% penicillin and streptomycin (Corning), 1% L-glutamine (Corning), and 0.05 mM β-mercaptoethanol (Sigma). All cells were maintained at 37°C in a 5% CO2 laboratory incubator subject to routine cleaning and decontamination. poly(I:C) was purchased from Invivogen. Antibodies were obtained from the following sources: mouse anti-NLRX1 (Jenny Ting laboratory), KSHV ORF45 (MA5-14769) (Thermo Scientific), Goat anti β-actin-HRP (1615) (Santa Cruz), KSHV K8.1alpha (SC-57889; Santa Cruz). The NLRX1 antibody was previously reported [12]. The pCIG2-Puro and pCIG2-NLRX1-FLAG plasmids were previously described [17]. The dRIG-I and MAVS expression plasmids were previously described [12]. pRL-CMV renilla vector was obtained from Promega. IFNβ promoter luciferase was a generous gift from Zhijian Chen, University of Texas Southwestern, Dallas. pUNO-IRF3sa was obtained from Invivogen.

### siRNA transfections and reactivation of KSHV in iSLK.219 and BCBL-1 cells

iSLK.219 cells were maintained as described above and were transfected using Lipofectamine RNAiMAX (Life Technologies) according to the manufacturer’s instructions. At 48 hours post-transfection, the medium was changed to DMEM containing 1% Pen-Strep, 10% FBS, and 0.2 μg/ml of doxycycline for reactivation [20]. BCBL-1 siRNA transfections were performed using Lonza nucleofector V kit according to the manufacturer’s recommendations (Lonza). BCBL-1 PEL cells were reactivated with 1 mM sodium butyrate (Sigma) and 25 ng/ml 12-O-tetradecanoyl-phorbol 13-acetate (TPA) (Sigma) where indicated. At 0, 24, 48 or 72 hours post-reactivation, cells and supernatant were collected. RNA was harvested from cells via the RNeasy Plus mini kit (Qiagen) for analysis of levels of viral transcripts. DNA was harvested from both cells and supernatant via DNeasy mini kit (Qiagen) for analysis of genome copy numbers. Protein from cells was harvested for WB analysis.

### RNA interference

Chemically synthesized siRNA duplexes were obtained from Dharmacon GE. ON-TARGETplus Non-targeting Control siRNAs #1 (D-001810-01); ON-TARGETplus NLRX1 siRNA (J-012926-10) Sequence: UCGUCAACCUGGUGCGCAA; ON-TARGETplus NLRX1 siRNA #2 (J-012926-12) Sequence: GUGCUGGGUUUGCGCAAGA; ON-TARGETplus TBK1 (29110) siRNA SMARTpool (L-003788-00) Sequences: (J-003788-08) AGAAGGCACUCAUCCGAAA; (J-003788-09) GAACGUAGAUUAGCUUAUA; (J-03788-10) UGACAGCUCAUAAGAUUUA; (J-003788-11) GGAUAUCGACAGCAGAUUA

### Quantitative Real-Time PCR

Total RNA was isolated by using RNeasy RNA extraction kit (Qiagen) and cDNA synthesis was performed using iScript cDNA Synthesis Kit (Bio-rad) according to manufacture protocols. Real-time PCR was performed using a ViiA 6 Real-Time PCR System. A SYBR green assay from Bio-rad was used for human *ifnb* as well as KSHV ORFs detection. Primers used for SYBR green qRT-PCR were:

KSHV *orf57* F: 5’-TGGACATTATGAAGGGCATCCTA-3’; R: 5’-CGGGTTCGGACAATTGCT-3’.

KSHV *orf36* F: 5’-TGCGTCCTCTTCCAGTGTTA-3’; R: 5’-GTCAGCAGAGTGTAGCCCAA-3’.

KSHV *virf1* F: 5’-CGTGTCCTTTGGTGAAACTG-3’; R: 5’-TCGGCATTATTTCGAGTACG-3’.

KSHV *k8*.*1* F: 5’-AAAGCGTCCAGGCCACCACAGA-3’; R: 5’-GGCAGAAAATGGCACACGGTTAC-3’.

Human *actin* F: 5’-AAGACCTGTACGCCAACACA-3’; R: 5’-AGTACTTGCGCTCAGGAGGA-3’.

Human *ifnb* F: 5’-AGTAGGGCGACACTGTTCGTG-3’; R: 5’-GAAGCACAACAGGAGAGCAA-3’.

Human *nlrx1* F: 5’-CCTCTGCTCTTCAACCTGATC-3’; R: 5’-CCTCTCGAAACATCTCCAGC-3’.

Human *tbk1* F: 5’- CCTCCCTAAAGTACATCCACG-3’; R: 5’- CAATCAGCCATCGTATCCCC-3’.

The relative amount of IFNβ, ORF57, ORF36 and K8.1 mRNA was normalized to actin RNA level in each sample and the fold difference between the treated and mock samples was calculated.

### JAK-STAT array

Total RNA was isolated from iSLK.219 or BCBL-1 cells by using RNeasy RNA extraction kit (Qiagen) and cDNA synthesis was performed using iScript^™^ cDNA Synthesis Kit (Bio-rad) according to the manufacturer’s protocols. Human JAK/STAT Signaling Primer Library was purchased from Realtimeprimers.com (Cat #: HJAK-I), which contains 88 primer sets directed against human JAK/STAT related genes and 8 housekeeping gene primer sets. Fold-Change (2^(- Delta Delta Ct)) is the normalized gene expression (2^(- Delta Ct)) in the Test Sample divided the normalized gene expression (2^(- Delta Ct)) in the Control Sample. Data were analyzed by GENE-E software (https://software.broadinstitute.org/GENE-E/).

### qPCR viral array

We used a real-time qPCR array to quantify all KSHV mRNAs. Briefly, 192 primer pairs were included to target multiple regions towards the 3’ end of each annotated ORF. Multiple reference genes for cellular transcripts were included for normalization. The array results in amplification reactions with similar efficiencies and annealing temperatures and thus allows us to directly compare the expression levels among different mRNAs. qPCR was plated in 384-well plates using the Tecan Freedom Evo liquid handling robot and cycled using Roche LightCycler 480, as previously described. A detailed, step-by-step protocol is available at http://www.med.unc.edu/vironomics/protocols.

### Statistical analysis

Statistical significance of differences in cytokine levels, mRNA levels, viral titers, and luciferase intensity in reporter assay were determined using Student’s t-test. * indicates P<0.05. ** indicates P<0.01.

## Supporting information

S1 Fig Overexpression of NLRX1 attenuates IFNβ and enhance KSHV gene expression upon reactivation. iSLK.219 cells were transfected with vector or NLRX1 plasmids for 48 hours and then treated with Dox for various time points. (A) qRT-PCR of *ifnb* in reactivated iSLK.219 cells. (B) qRT-PCR of *orf57* in reactivated iSLK.219 cells. (C) qRT-PCR of *nlrx1* in reactivated iSLK.219 cells. (D-F) HEK293T cells were co-transfected as indicated with NLRX1 plasmid, IFNβ-luc (50ng) and pRL-CMV (10ng) with 50ng of dRIG-I (D), MAVS (E) or IRF3(SA) (F). 48 hours later, luciferase activity were monitored.
(EPS)

S2 Fig Knockdown of NLRX1 attenuates KSHV gene expression upon reactivation. iSLK.219 cells were transfected with NS, NLRX1 or NLRX1 #2 siRNA for 48 hours and then treated with Dox for various time points. (A-B) Whole well GFP (A)/RFP (B) intensities were monitored and quantitated by a Clariostar plate reader. (C-E) qRT-PCR of *virf1* (C), *k8*.*1* (D) or *nlrx1* (E) in reactivated iSLK.219 cells.
(EPS)

S3 Fig Knockdown of NLRX1 attenuates KSHV gene expression upon reactivation without affecting Dox-induced RTA expression. iSLK.219 cells were transfected with NS or NLRX1 siRNA for 48 hours and then treated with Dox for various time points. (A-C) qRT-PCR of *nlrx1* (A), *virf1* (B) or *k8*.*1* (C) in reactivated iSLK.219 cells. iSLK.RTA cells were transfected with NS or NLRX1 siRNA for 48 hours and then treated with Dox for 24h (D-E) qRT-PCR of *rta* (D) or *nlrx1* (E) in reactivated iSLK.RTA cells.
(EPS)

S4 Fig Comparison of JAK/STAT related genes activated by knockdown of NLRX1 without reactivation of KSHV in iSLK.219 cells. iSLK.219 cells were transfected with NS, NLRX1 or NLRX1 #2 siRNA for 48 hours. RNA was harvested from iSLK.219 and made into cDNA. (A-B) Regulation of JAK/STAT related genes in siNLRX1 (A) or siNLRX1 #2 (B) were compared to the siNS group. (C-D) Comparison of upregulated genes (C) or downregulated genes (D) in two experiments.
(EPS)

S5 Fig Poly I:C transfection activates IFNβ and inhibits KSHV gene expression upon reactivation. iSLK.219 cells were transfected with NS or NLRX1 siRNA for 48 hours and then treated with Dox for various time points. (A-B) Whole well GFP (A)/RFP (B) intensities were quantitated by a Clariostar plate reader. (C-F) qRT-PCR of *ifnb* (C), *orf57* (D), virf1 (E) or k8.1 (F) in reactivated iSLK.219 cells.
(EPS)

S6 Fig Knockdown of TBK1 enhances KSHV gene expression upon reactivation. iSLK.219 cells were transfected with NS, or TBK1 siRNA for 48 hours and then treated with Dox for various time points. (A-D) qRT-PCR of *tbk1* (A), orf57 (B), *virf1* (C) or *k8*.*1* (D) in reactivated iSLK.219 cells.
(EPS)

S7 Fig Knockdown of NLRX1 by a different NLRX1 siRNA enhances KSHV gene expression in reactivated BCBL-1 cells. BCBL-1 cells were transfected with NS or NLRX1 #2 siRNA for 48 hours and then treated with Dox for various time points. (A-D) qRT-PCR of *nlrx1* (A), orf57 (B), *virf1* (C) or *k8*.*1* (D) in reactivated BCBL-1 cells.
(EPS)

S1 Table iSLK.219 JAK/STAT pathway genes analysis in microarray. (XLSX)

S2 Table BCBL-1 JAK/STAT pathway genes analysis in microarray. (XLSX)
